# Nickel Acetate-Assisted Graphitization of Porous Activated Carbon at Low Temperature for Supercapacitors With High Performances

**DOI:** 10.3389/fchem.2022.828381

**Published:** 2022-03-02

**Authors:** Xiaohui Zhang, Zhian Qiu, Qingyu Li, Libo Liang, Xiaofei Yang, Shaorong Lu, Dinghan Xiang, Feiyan Lai

**Affiliations:** ^1^ Guangxi Hezhou Guidong Electronic Technology Co. Ltd, Hezhou, China; ^2^ Guangxi Key Laboratory of Calcium Carbonate Resources Comprehensive Utilization, Hezhou University, Hezhou, China; ^3^ College of Materials Science and Engineering, Guilin University of Technology, Guilin, China; ^4^ Guangxi Key Laboratory of Information Materials, Guilin University of Electronic Technology, Guilin, China; ^5^ Guangxi Key Laboratory of Low-Carbon Energy Materials, Guangxi Normal University, Guilin, China

**Keywords:** supercapacitors, activated carbons, catalytic graphitization, pore-forming agent, biomass

## Abstract

Catalytic graphitization opens a route to prepare graphitic carbon under fairly mild conditions. Biomass has been identified as a potentially attractive precursor for graphitic carbon materials. In this work, corn starch was used as carbon source to prepare hollow graphitic carbon microspheres by pyrolysis after mixing impregnation with nitrate salts, and the surface of these carbon microspheres is covered with controllable pores structure. Under optimal synthesis conditions, the prepared carbon microspheres show a uniform pore size distribution and high degree of graphitization. When tested as electrode materials for supercapacitor with organic electrolyte, the electrode exhibited a superior specific capacitance of 144.8 F g^−1^ at a current density of 0.1 A g^−1^, as well as large power density and a capacitance retention rate of 93.5% after 1,000 cycles in galvanostatic charge/discharge test at 1.0 A g^−1^. The synthesis extends use of the renewable nature resources and sheds light on developing new routes to design graphitic carbon microspheres.

## Introduction

Supercapacitors, as a kind of promising energy storage device with high power density and long service life, have excited great interest owing to their potential application in portable electronics and electric vehicles (Qin et al., 2021; Reece et al., 2020; Yang L et al., 2021; Zhu et al., 2019). Based on different energy storage mechanisms, supercapacitors are mainly divided into two categories: faradic pseudocapacitors, in which transition metal oxides and conducting polymers are used as active materials, and electrical double-layer capacitors (EDLCs), in which carbon-based materials are often used as active materials (Adhikari et al., 2020; Liu T et al., 2020; Say et al., 2020). The energy storage mechanism of EDLCs depends on the attraction of positive and negative charges at the interface of electrode and electrolyte, enabling high power density and ultralong cycling life of EDLCs (Fleischmann et al., 2020; Pai et al., 2021; Pourhosseini et al., 2021). Activated carbons (ACs) are the most commonly used electrode material for EDLCs (Zhang et al., 2015; Zhang et al., 2018; Zhang et al., 2019), which possess high surface area, good electrochemical stability, and affordable cost (Heidarinejad et al., 2020; Lv et al., 2021; Wu et al., 2021).

However, high resistance of ACs increases internal resistance and reduces specific capacitance at high current density of supercapacitors (Li et al., 2021; Zhang et al., 2021; Fan et al., 2021). In addition, the high resistance easily cause heat generation. Graphitization can improve conductivity of ACs to a certain extent but requires harsh experimental conditions which hamper large-scale preparation (Jiang et al., 2020; Jiang et al., 2021a). Hence, it is necessary to keep working to search sustainable methods suitable for large-scale production. Catalytic graphitization opens a route to prepare graphitic carbon under fairly mild conditions. The conversion temperature of amorphous carbonaceous materials to graphitic carbon can be reduced to less than 800°C (Jiang et al., 2021b; Lam et al., 2021; Yang L et al., 2021). A variety of transition metal catalysts are commonly used, including Ni (Yang L et al., 2021), Co (Liu T et al., 2020), and Fe (Semeniuk et al., 2021). The carbon source is pyrolyzed after mixing and impregnating transition metal salts.

Biosource-derived carbon precursors are attractive and widely used in fabrication of ACs electrodes, due to environment, sustainability, and cost concerns (Liu Z. et al., 2020; Ait et al., 2021; Baig et al., 2021; Raj et al., 2021; Wang et al., 2021). For example, as a typical low-cost and renewable biomass material, various starches have been applied widely in the preparation of porous carbon materials by enzymatic hydrolysis (Wang et al., 2020; Yin et al., 2020). Herein, we proposed a facile approach to fabricate graphitic porous carbon microsphere utilizing corn starch as carbon precursor by pyrolysis after mixing and impregnating Ni(CH_3_COO)_2_ as graphitization catalyst. The surface of hollow spherical particles is covered with pores increasing ion transferring as well as the enhanced electrical conductivity improves the rate capability. When tested as electrode materials for supercapacitor with organic electrolyte, the electrode exhibits a superior specific capacitance of 144.8 F g^−1^ at 0.1 A g^−1^ and good large power density and a capacitance retention rate of 93.5% after 1,000 cycles at 1.0 A g^−1^. In addition, the simple synthesis method develops a new route to prepare graphitic carbon microspheres.

## Experimental Section

### Material Synthesis

The suspension of corn starch (100 g) mixed with 100 ml Ni(CH_3_COO)_2_ solution (0.1 mol L^−1^) was stirred for 10 h and freeze-dried for 24 h. The dried mixture was then heated at 210°C for 20 h and carbonized at 450°C for 3 h. The KOH saturated solution was added by a stoichiometric proportion of 5: 1 to mix the starch-derived carbon. The mixture was transferred to nickel crucible and activated at 850°C for 2 h under Ar atmosphere with a heating rate of 5°C min^−1^. The product was firstly impregnated in 0.1 M HCl and then washed by distilled water. The final product is marked as Ni-AC. The contrast sample signed as AC was prepared through the same experiment process to the Ni-AC, but the Ni(CH_3_COO)_2_ was not added.

### Materials Characterization

The morphology of the as-prepared Ni-AC and AC was observed by field emission scanning electron microscopy (SEM, Philips, FEI Quanta 200FEG). The component of carbon materials was measured by energy dispersive X-ray spectroscopy (EDS, EDAX JENSIS60S). The analysis of phase composition was investigated by powder X-ray diffraction (XRD, Rigaku, D/max 2500 v/pc) with Cu-Ka1 radiation. The Nitrogen adsorption/desorption isotherms and pore size distribution were tested by an automatic volumetric sorption analyzer at 77 K (Micromeritics, SA3100, United States).

### Electrochemical Testing

The product powder was mixed with binders carboxymethyl cellulose (CMC), polymerized styrene butadiene rubber (SBR), and conductive agents SP, which were dispersed in aqueous solvent with a weight ratio (activated materials: CMC: SP: SBR = 81: 6: 10: 3). The stirred slurry was pasted onto Al foil and dried at 80°C in vacuum. The electrode film was punched in a specified diameter of 18 mm. The foil was equipped into a button battery model (CR 2032), in which the electrolyte is 1 mol L^−1^ C2H5) 4NBF4/PC and the separator is organic special diaphragm (NKK, 4020).

The electrochemical tests were carried out in a three-electrode cell on an IM6 electrochemical workstation (Zahner-Elektrik, Germany). Cyclic Voltammetry (CV) was scanned from 20 to 200 mV s^−1^, and the voltage window is between 0 and 2.7 V in a two-electrode cell. Galvanostatic charge-discharge tests were performed with the current densities at 0.1, 0.5, and 1.0 A g^−1^ on LAND system (CT 2001A, China). Electrochemical impedance spectra (EIS) were acquired from 10 to 1 MHz with an open circuit at an amplitude of 5 mV.

## Results and Discussion

**FIGURE 1:**
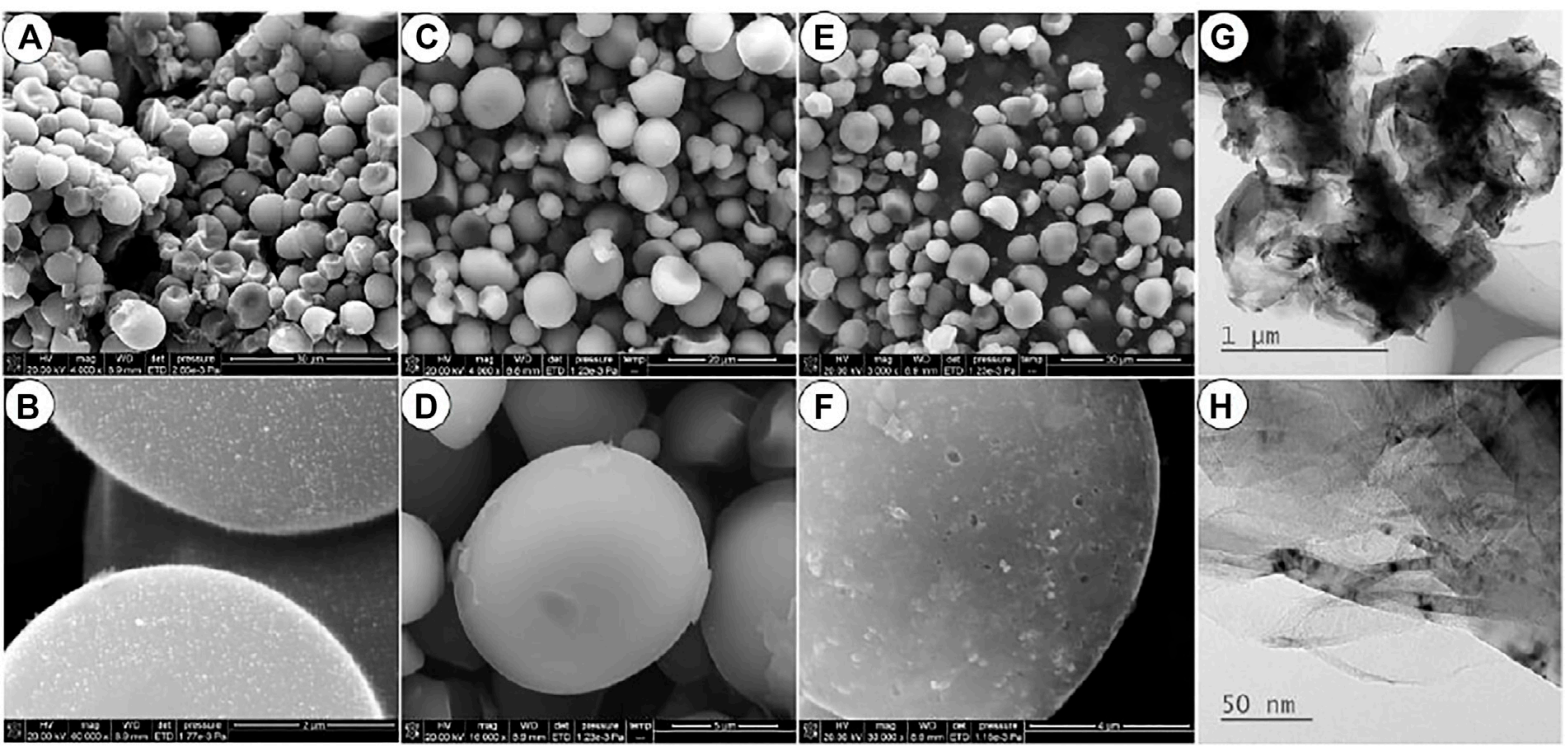
The SEM of carbonized precursor **(A,B)**, AC **(B,D)** and Ni-AC **(E,F)**, and the TEM of the Ni-AC **(G,H)**.

Figures 1A,B show the SEM images of the carbonized precursor of the prepared Ni-AC, the precursor presents a similar spherical shape with a particle size of 5–10 μm. Some hemispherical particles indicate that these spherical particles have a hollow structure. The tiny Ni-containing particles are dispersed uniformly on the surface from Figure 1B. The content of the Ni element in the Ni-AC composite is 0.13% by the ICP analysis. After activation, both of the prepared active carbon products keep the original spherical shape of the precursor as well as the particle size, as shown in Figures 1C,E. The spherical shape can increase the tap density and lead to an enhanced volumetric capacity (Marriam et al., 2020). Moreover, the good liquidity of spherical materials is in propitious to preparing electrode (Ge et al., 2021; Xie et al., 2021). After activation, both the particles of the two samples keep spherical shape and size as shown in Figures 1C,D. However, some detailed morphology difference can be observed in the larger magnification of Figures 1D,F. Many holes and pits appear on surface of the Ni-AC while a smooth surface is left for the AC sample carbonized without Ni(CH_3_COO)_2_. It can be inferred that these surface defects are left by the dissolution of the tiny Ni-containing particles. The size of the Ni particles is less than 5 nm measured in the provided TEM images (Figures 1G,H).

**FIGURE 2:**
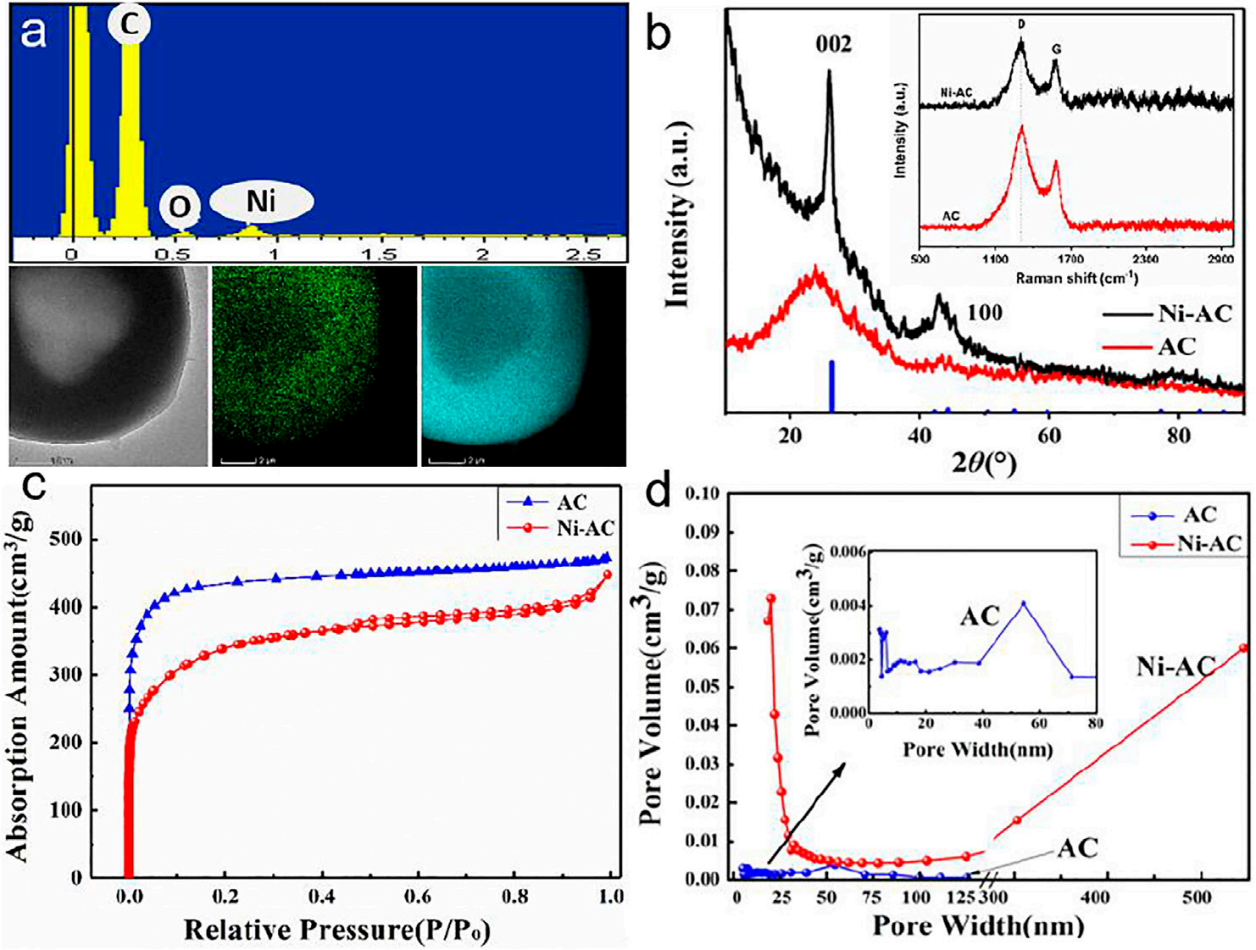
EDS spectrum and mapping of the carbonized precursor with Ni(CH_3_COO)_2_ **(A)**, XRD patterns and Raman spectra **(B)**, nitrogen adsorption/desorption isotherms **(C)**, and pore size distribution curves **(D)** of the Ni-AC and AC.

EDS mapping images show that the Ni element is uniformly distributed throughout the entire surface of the spheres, suggesting the tiny particles are Ni-containing composite, usually nickel carbide reduced from Ni(CH_3_COO)_2_ (Figure 2A). Figure 2B shows the XRD patterns of the final products. The diffraction peaks at 2θ = 24° for both the Ni-AC and AC corresponding to the pattern of graphite. The Ni-AC has a sharper diffraction peak, while the corresponding *I*_D_/*I*_G_ value in Raman spectra is 1.50 which is less than that of the AC (1.55). These results reveal a higher degree of graphitization of the Ni-AC due to the addition of Ni element playing a role in catalyzing graphitization. The detailed pore textural characteristics of the samples were analyzed by Nitrogen sorption technique. The adsorption/desorption isotherms plots and pore size distribution are shown in Figures 2C,D. Both the two samples present typical characteristics of ACs. In addition, the curve of the Ni-AC exhibits a significant hysteresis loop, indicating many mesopores are dominated in the pores. The catalytic role of Ni(CH_3_COO)_2_ improves the graphitization reaction and creates micropores, which facilitate mobility of electrolyte ions, leading to an excellent charge/discharge rate capability (Liu T et al., 2020; Pang et al., 2020). Moreover, the Ni-AC spheres have a narrow distribution at approximately 25 nm, meaning the Ni-AC has uniform pore size distribution compared to the AC with a broad size distribution at around 5–50 nm. The BET surface areas of the AC and Ni-AC are 1912 and 1775 m^2^/g, respectively. Some results reveled the electrode materials would maximize inner quality if the pore size distribution is closed to the size of electrolyte molecules, and more larger or smaller pores would cause a significant drop in capacitance (Zhang et al., 2008).

**FIGURE 3:**
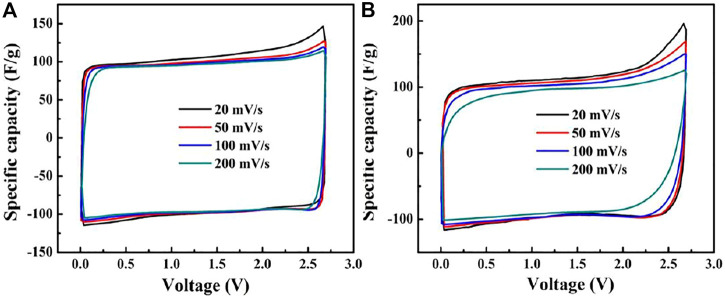
The CV curves of Ni-AC **(A)** and AC **(B)** in different scan rates.

Figures 3A,B show the CV curves of the Ni-AC and AC, respectively. The curves of the Ni-AC show a typical double-layer capacitance behavior and are of good rectangular shapes even at high scanning ratios, suggesting the sample is absolutely capable to capacitor materials. The specific capacitance is estimated by the following equation: *C*=*I*/*r*, where *I* is current density and *r* is potential scan. The specific capacitance of the Ni-AC can reach 110 F g^−1^ at 20 mV s^−1^. As the scanning ratio increases to 200 mV s^−1^, the capacitance decreases only 9%, while the AC lefts 18.97%. It is the interface rather than in the bulk that take place in charges collection for EDLC, so the interface between the electrode and the electrolyte plays a pillar role in the capability. Meanwhile, the charges collection induces only physical absorption instead of occurring faradic effects, and this kind of adsorption performance means no redox peaks on CV curves. Obviously, both the curves in Figures 3A,B have no redox peaks, meaning that the two materials perform physical absorption. The curve shapes of the Ni-AC electrode exhibit less shape change than the AC with the scan rate increasing, suggesting smaller polarization associated with higher conductivity due to improved graphitization degree.

**FIGURE 4:**
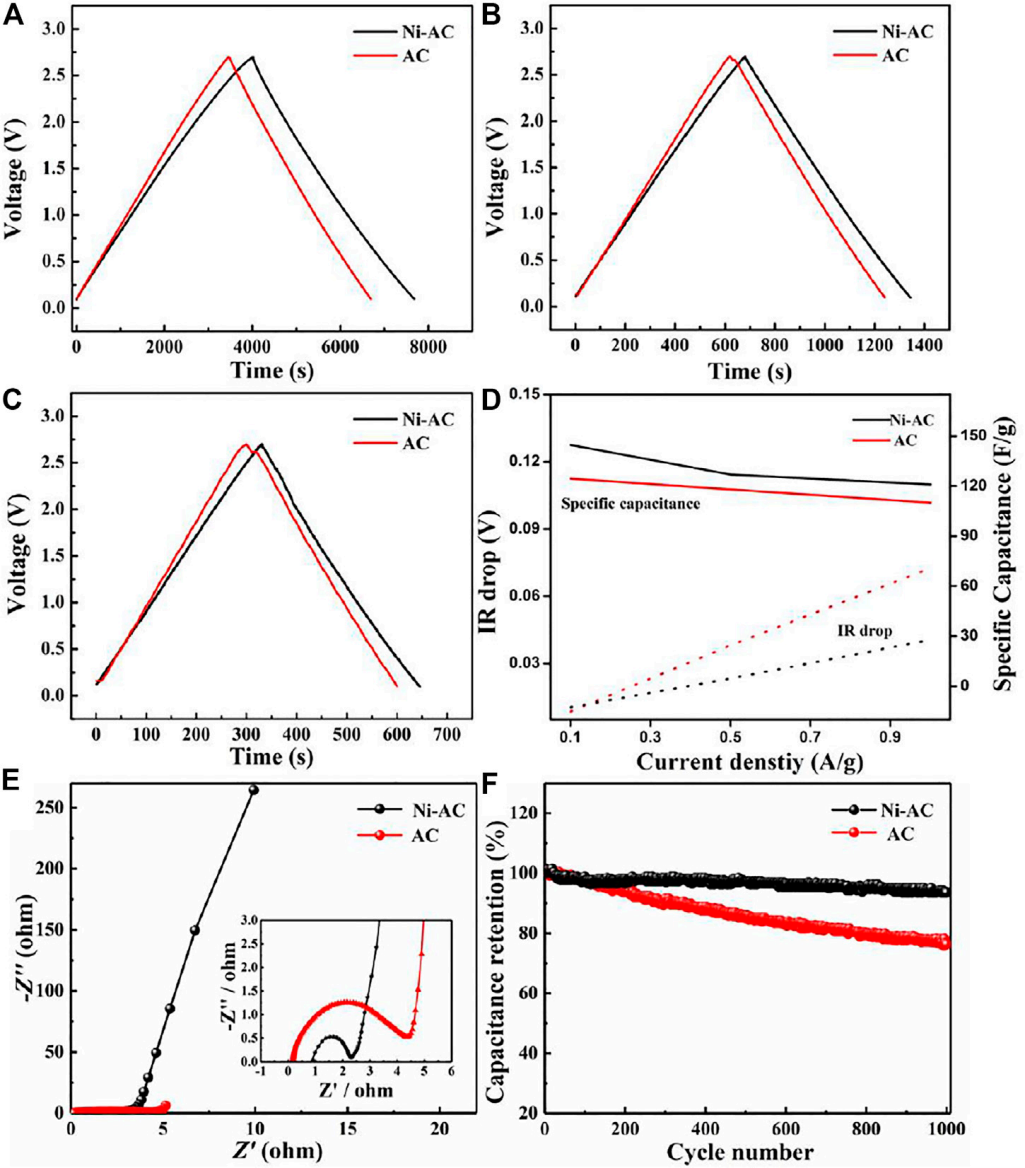
The galvanostatic charge-discharge curves at 0.1 **(A)**, 0.5 **(B)**, and 1 A g^−1^ **(C)**, and the specific capacitance and IR drop under different current densities **(D)**, Nyquist plots **(E)**, and cycle performance **(F)** of the Ni-AC and AC electrodes.

Galvanostatic charge-discharge curves the Ni-AC and AC electrodes at various current densities as depicted in Figures 4A–C. The specific capacitance was calculated according to the following equation: *C*=*I*/(∆*V*/∆*t*), where *I* and ∆*V*/∆t are current density and discharge slope after IR drop, respectively. The specific capacitance of the Ni-AC is 145, 127, and 121 F g^−1^ at 0.1, 0.5, and 1 A g^−1^, while the AC delivers 124, 118, and 110 F g^−1^ at the same current densities. The galvanostatic charge-discharge curves of the Ni-AC maintain a good isosceles triangle even at a high current density of 1 A g^−1^. All the results indicate that the Ni-AC has good capacitance behavior and endures high current charge-discharge capability associated with a uniformed mesopores distribution. Generally, the specific capacitance is in opposite proportion to the increasing current density due to IR drop, which is mostly caused by higher resistance of ion transfer. The IR drop depends on the overall resistance of a cell, determined from the voltage decrease at the start of the galvanostatic discharge curves (Teng et al., 2001). As shown in Figure 4D, the amplitude of IR drop for both electrodes raises along with current density increasing. The IR drop of the Ni-AC is lower than that of the AC at the same currents, illustrating the ion-transfer resistance of the AC electrode is higher than that of the Ni-AC electrode. This conclusion can also be confirmed by the Nyquist plots of the electrodes, as shown in Figure 4E. Both the Nyquist plots of the two electrodes contain one semicircle in high frequency region corresponding to charge transform resistance at electrolyte/electrode interface and an upward line in low frequency region associated with ion diffusion in electrolyte (Adhikari et al., 2020; Gurten and Aktas, 2020; Pang and Wang, 2021). The Ni-AC electrode has smaller charge transferring resistance than that of the AC, which contributes to the enhanced graphitization degree boosting conductivity. Slightly, there is no big difference between the Ni-AC electrode and AC electrode, confirming both electrodes have low electrolyte ion diffusion resistance and ideal capacitor behavior. Figure 4F shows the cycle performance of the Ni-AC and AC electrodes. The capacitance of the Ni-AC is almost unchanged along with the increasing cycle number, while the AC capacitance retention shows a downward tendency. After calculation, the capacitance retention of the Ni-AC is up to 93.5%, much higher than that of the AC electrode (76.4%) after 1,000 cycles, indicating that the Ni-AC electrodes have a better cycle performance and stability.

## Conclusion

Catalytic graphitization of corn starch derived carbon microspheres was achieved under Ni(CH_3_COO)_2_ using nitrate salts as catalyst. In the prepared graphitic hollow carbon microspheres in this work, corn starch shows porous structure on the surface. The active carbons are of well-developed mesoporous structure, uniformed pore size distribution, and a high level of graphitization, which sufficiently improve the capacity performance and rate capability in supercapacitor. Under optimal synthesis conditions, the electrochemical investigations of electrode are of 114 F g^−1^ capacitance, good capacitance behavior, low level of ion-transfer resistance, and good cycle stability.

## Data Availability

The original contributions presented in the study are included in the article/supplementary material, and further inquiries can be directed to the corresponding authors.
